# Editorial: Exploring goal-directed behavior through creativity: perspectives from psychology, neuroscience, and psychiatry

**DOI:** 10.3389/fpsyg.2024.1407344

**Published:** 2024-04-19

**Authors:** Chong Chen, Hadi Moradi, Leila Kashani Vahid, Radwa Khalil

**Affiliations:** ^1^Division of Neuropsychiatry, Department of Neuroscience, Yamaguchi University Graduate School of Medicine, Ube, Japan; ^2^School of Electrical and Computer Engineering, University of Tehran, Tehran, Iran; ^3^Department of Psychology, Azad University, Science and Research Branch, Tehran, Iran; ^4^School of Business, Social, and Decision Sciences, Constructor University, Bremen, Germany

**Keywords:** goal-directed behavior, creativity, individual differences, mindset, transcranial direct current stimulation, creative ideation, response inhibition, risk-taking

Goal-directed behavior and creativity are interconnected aspects of human cognition. Goal-directed behavior involves setting and pursuing goals effectively, identifying desired outcomes, formulating plans, and executing actions (Toba et al., 2023; De Houwer et al., 2024). It is studied in psychology and neuroscience to understand how individuals prioritize tasks, allocate resources, and adapt their behavior, and has been found to be closely related to general intelligence (Duncan et al., 2008). On the other hand, creativity involves generating valuable ideas, solutions, or products, driving innovation, and problem-solving in various fields (for a review, see Abraham, 2018; Ivcevic et al., 2023). The relationship between these two is complex and dynamic, with goals motivating creativity and creativity expanding the range of possible goals and strategies. Understanding this dynamic relationship is crucial for understanding human cognition and behavior in various contexts, enabling the development of strategies to foster creativity and innovation. Despite considerable advancements in creativity research from psychological perspectives, the core cognitive and neural mechanisms underlying creative thinking require further investigations using integrative approaches, which also require cross-talk between different disciplines. This integration is crucial, particularly in reflecting the dynamic nature of creativity (Agnoli, 2024). This Research Topic, comprising five articles, aims to tackle some of these crucial key questions, striving to deepen our understanding of the neurocognitive and computational foundations of creativity, as well as its facilitation in the context of the workplace and daily life.

Khalil et al. utilized a brain stimulation technique called transcranial direct current stimulation (tDCS) to examine the neuromodulatory signatures linked to creative ideation. This study evaluated how an individual's mindset influences response inhibition (RI) and divergent thinking (DT) using tDCS. It included 40 undergraduate students between the ages of 18 and 23. The results of the Alternative Uses Task (AUT) showed that the levels of mindset changed the stimulation conditions, leading to an increase in RI for fluency and flexibility but not originality. Interestingly, growth mindsets had the reverse effect on DT, resulting in decreased fluency but increased flexibility. According to this study, it is essential to consider cognitive status and control functions to understand how tDCS changes the brain during ideational processes. This paper offered a new perspective on the moderating function that mindset (in the sense of cognitive status and, more specifically, the growth mindset) plays in psychological development.

In a thoroughly designed experiment, Harada (a) explored the impact of group dynamics on the relationship between risk-taking and divergent thinking. Participants engaged individually, in dyadic pairs, or in triadic groups. Triadic group participants outperformed others on the AUT. Risk attitudes were assessed using a two-armed bandit reinforcement learning task combined with a Q-learning computational model. In individual settings, risk aversion in gains and risk-seeking in losses negatively correlated with AUT composite scores, whereas loss aversion positively correlated with AUT composite scores. Risk attitudes were unrelated to AUT composite scores in dyadic pairs, while loss aversion was negatively correlated with them. In triadic groups, risk aversion in gains positively correlated with AUT composite scores, while risk-seeking in losses negatively correlated with them; loss aversion showed no relevance. These findings underline the value of considering group dynamics and individual risk attitudes in fostering creativity within organizational contexts.

Using the same computational framework, in another study, Harada (b) investigated insight problem-solving, a facet of convergent thinking. Loss aversion is inversely correlated with performance on the 9-dot problem. For second-time tasks, loss aversion negatively correlated with performance on both the 8-coin problem and the 9-dot problem, suggesting the importance of accepting losses for successful insight problem-solving. This aligns with recent evidence suggesting excessive loss aversion's maladaptive nature and its association with poor psychological growth and various neuropsychiatric issues (Koan et al., 2021).

Fürst and Grin investigated the association between multilingualism and creativity. Utilizing a latent variable model, they found that multilingualism and multicultural experiences positively relate to creativity personality, which further contributes to creative activities across various domains, including music, writing, the arts, inventions, and science. This finding is consistent with a growing body of evidence that links multilingualism and multicultural experiences to creative thinking. This includes enhanced divergent thinking skills (Kharkhurin et al., 2023) and superior abilities in appreciating creative metaphors (Werkmann Horvat et al., 2021).

In a review article, Liu discussed the crucial role of feedback valence in team creativity. He suggested that accurate positive feedback provides strategic information and fosters employee interest in the topic. Conversely, although negative feedback may affect employee self-esteem, it is pivotal for performance improvement. Liu also highlighted a recent study by Hoever et al. (2018) that suggested an interaction between feedback valence and informational diversity in team creativity. Hoever et al. (2018) found that negative feedback fosters team creativity through elaboration in teams with diverse information. Elaboration involves sharing, discussing, or integrating members' informational resources, which enriches the creative process by incorporating diverse perspectives and knowledge. Conversely, positive feedback enhanced creativity through generative processing in informationally homogeneous teams. Generative processing occurs when team members stimulate each other to generate novel and useful insights. Liu further explored recent advances and underscored important and significant limitations that need to be addressed in future research.

Furthermore, recent studies have suggested a myriad of activities that may enhance creativity, including physical activity (Matsumoto et al., 2022; Kawashima et al., 2024), immersing oneself in nature (Atchley et al., 2012), and listening to uplifting music (Ritter and Ferguson, 2017, for reviews, see Khalil and Demarin, 2023; Chen, 2024). However, a comprehensive understanding of the factors influencing creativity and effective strategies to promote it in everyday settings, such as the workplace, requires deeper investigation.

In conclusion, the examination of creativity within various contexts, as showcased in the five articles on this Research Topic, underlines its multifaceted nature and the complexity of its determinants. From analyzing neuromodulatory signatures to understanding group dynamics and feedback mechanisms, each study contributes valuable insights into the mechanisms underlying creative thinking and its facilitation in diverse settings. As we continue to connect the dots between brain processes and the processes of creativity, it becomes increasingly clear that fostering innovation requires a comprehensive understanding of cognitive, neural, social, and environmental factors. By integrating knowledge from psychology, neuroscience, and other disciplines, we can pave the way for more effective strategies to nurture creativity in everyday life, ultimately driving progress and innovation across fields and industries.

## Author contributions

CC: Writing – original draft, Writing – review & editing. HM: Writing – review & editing. LK: Writing – review & editing. RK: Writing – review & editing.
